# A new mutation in *BFSP2* (G1091A) causes autosomal dominant congenital lamellar cataracts

**Published:** 2008-10-24

**Authors:** Xu Ma, Fei-Feng Li, Shu-Zhen Wang, Chang Gao, Meng Zhang, Si-Quan Zhu

**Affiliations:** 1 Graduate School, Peking Union Medical College, Beijing, China; 2 Department of Genetics, National Research Institute for Family Planning, Beijing, China; 3 WHO Collaborative Center for Research in Human Reproduction, Beijing, China; 4 Beijing Tongren Eye Center, Capital Medical University, Beijing, China

## Abstract

**Purpose:**

We sought to identify the genetic defect in a four-generation Chinese family with autosomal dominant congenital lamellar cataracts and demonstrate the functional analysis with biosoftware of a candidate gene in the family.

**Methods:**

Family history data were recorded. Clinical and ophthalmologic examinations were performed on family members. All the members were genotyped with microsatellite markers at loci considered to be associated with cataracts. Two-point LOD scores were calculated by using the Linkage Software after genotyping. A mutation was detected by using gene-specific primers in direct sequencing. Wild type and mutant proteins were analyzed with Online Bio-Software.

**Results:**

Affected members of this family had lamellar cataracts. Linkage analysis was obtained at markers D3S2322 (LOD score [Z]=7.22, recombination fraction [θ]=0.0) and D3S1541 (Z=5.42, θ=0.0). Haplotype analysis indicated that the cataract gene was closely linked to these two markers. Sequencing the beaded filament structural protein 2 (*BFSP2*) gene revealed a G>A transversion in exon 5, which caused a conservative substitution of Arg to His at codon 339 (P.R339H). This mutation cosegregated with the disease phenotype in all affected individuals and was not observed in the unaffected family members or in 100 normal, unrelated individuals. Bioinformatic analyses showed that a highly conserved region was located around Arg339. Data generated with Online Bio-Software revealed that the mutation altered the protein’s hydrophobicity, hydrophobic moment, and chaperone and regulation activities.

**Conclusions:**

This is the first reported case of a congenital lamellar cataract phenotype associated with the mutation of Arg339His (P.R339H) in *BFSP2*. It highlights the physiologic importance of the beaded filament protein and demonstrates a possible mechanism of action for the mutant gene.

## Introduction

Hereditary congenital cataract is a clinically and genetically heterogeneous lens disease responsible for a significant proportion of visual impairment and blindness in childhood [1,2]. It can occur in an isolated fashion or as one component of a multi-system disorder. Non-syndromic congenital cataracts have an estimated incidence of 1–6 per 10,000 live births [3-6], and at least one-third of the cases are familial.

From the first description of the cosegregation of inherited cataracts with the Duffy blood group locus [7], more than 30 loci have been mapped through linkage analysis and 19 genes have been characterized. Among the 19 genes, 10 genes encode crystallins (*CRYAA*, *CRYAB*, *CRYBA1/A3*, *CRYBA*, *CRYBB1*, *CRYBB2*, *CRYBB3*, *CRYGC*, *CRYGD*, *CRYGS*), three encode membrane transport proteins (*MIP*, *GJA3*, *GJA8*), two encode a cytoskeletal protein (*BFSP1*, *BSFP2*), three encode transcription factors (*HSF4*, *MAF*, *PITX3*), and one encodes a lens intrinsic membrane protein (*Lim2*) [8-12].

Lens-specific beaded filament structural protein–2 (BFSP2, also called phakinin, CP47, CP49, and LIFL-L [OMIM 603212]) has been mapped to human chromosome 3q21-q25 with the use of human-rodent somatic cell hybrids [13]. BFSP2 is one of the cytoskeletal proteins in the lens, which sustains lens cell morphology and participates in lens cell motility, and is very important by functioning in the maintenance of transparency of the lens.

We know from the previous report that *BFSP1* and *BFSP2* mutations can lead to congenital cataracts. But, so far, just one paper has reported that a *BFSP1* mutation causes autosomal recessive juvenile onset cataract [12]. There are five reported *BFSP2* mutations that were found to induce formation of autosomal dominant congenital cataract. These mutations were mainly in exon 2; clinical types of cataracts induced by *BFSP2* gene mutations are polymorphism cataracts, nuclear cataracts, cortical opacity Star cataracts, and gill-like cataracts etc. [14-18]. We first reported mutations in exon 5 of *BFSP2*, which can cause autosomal dominant congenital cataract, and *BFSP2* mutations can also cause congenital lamellar cataracts [14-18].

Lamellar cataracts are a common form of congenital cataract. *MIP* [19], *CRYAB* [20], *CRYBA1* [21], and *HSF4* [22] mutations can cause this. More recently, Jamie et al. [23] described a three-generation pedigree with X-linked congenital lamellar cataract. In this study, we report a four-generation Chinese family with congenital lamellar cataracts. Linkage analysis mapped the disease gene to 3q21–25, and a missense mutation (c.1091G→A) in *BFSP2* was identified in this family, which resulted in the substitution of Arg339His (P.R339H) in BFSP2. Analysis of the wild type and mutant proteins suggested that the increased hydrophobicity with the decreased hydrophobic moment of the mutant protein altered regulation activities of the BFSP2 protein and α-crystallin assemblies. These alterations may be the cause of congenital lamellar cataracts.

## Methods

### Clinical evaluation and DNA specimens

A four-generation family with non-syndromic congenital cataracts was recruited at the Beijing Tongren Eye Center (Capital Medical University, Beijing, China). Informed consent was obtained from each participant as consistent with the Declaration of Helsinki. The phenotype was documented by slit lamp photography. Genomic DNA was extracted from peripheral blood leukocytes using standard protocols.

### Genotyping

Polymerase chain reactions (PCRs) were performed with microsatellite markers close to candidate loci associated with autosomal congenital cataracts. PCR products from each DNA sample were separated from a 6% polyacrylamide gel and were analyzed. Pedigree and haplotype data were performed with Cyrillic (version 2.1) software. Exclusion analysis was performed by allele sharing in affected individuals.

### Linkage analysis

A two-point linkage was calculated with the LINKAGE (version 5.1) package. The cataracts in this family were analyzed as an autosomal dominant trait with full penetrance and a gene frequency of 0.001. The allele frequencies for each marker were assumed to be equal in both genders. The marker order and distances between the markers were taken from the NCBI database.

### DNA sequencing

Individual exons of the *BFSP2* cluster were amplified by PCR using primer pairs shown in Table 1 [18]. The PCR products were sequenced on an ABI3730 Automated Sequencer (PE Biosystems, Foster City, CA).

**Table 1:** Primer sequences for *BFSP2*.

**Gene (Exon)**	**Forward Primers (5′→3′)**	**Reverse Primers (5′→3′)**
*BFSP2* (1a)	AATGCACAAACCCAAATGGT	AGGCCCTGSSGACACT
*BFSP2* (1b)	GAGAGGCGAGTGGTAGTGGA	GGCCTCAGCCTACTCACAAC
*BFSP2* (2)	TGCAGACAGAGCATTTCCAC	GAGGGGTGTGAGCTGGATAA
*BFSP2* (3)	GCTGCAATTGCCTTCATTTT	GGGTAACCTGACCCAACTTCA
*BFSP2* (4)	TCTGTGAAGCCTGTGTCTGG	CCCGGCCTCAATTATTCTTT
*BFSP2* (5)	ACCCAGGAGGAGGAGGTTGT	GGGAATCCCCTGGAAACTAA
*BFSP2* (6)	GGGGAATAGTCCAGGCTACC	ATGGGTGCCTATGTGAGAGGG
*BFSP2* (7)	TTGTTCCAAAGGCCAGATTC	CACTCAAGGGAATCCTTCCA

### Denaturing high performance liquid chromatography

Denaturing high performance liquid chromatography (DHPLC) was used to screen the mutation identified in the patients, family members, and 100 normal control subjects in exon 5 of *BFSP2* by using a commercial system (Wave DHPLC; Transgenomic, San Jose, CA).

### Analysis of protein models with biosoftware

The tertiary structure of the protein is highly conserved. Both mutant and wild type versions of the protein structure were predicted and analyzed using the Swiss-model software (version 3.5), BioEdit software, Phyre software (version 0.2), and Radar.

## Results

### Clinical data

**Figure 1:**
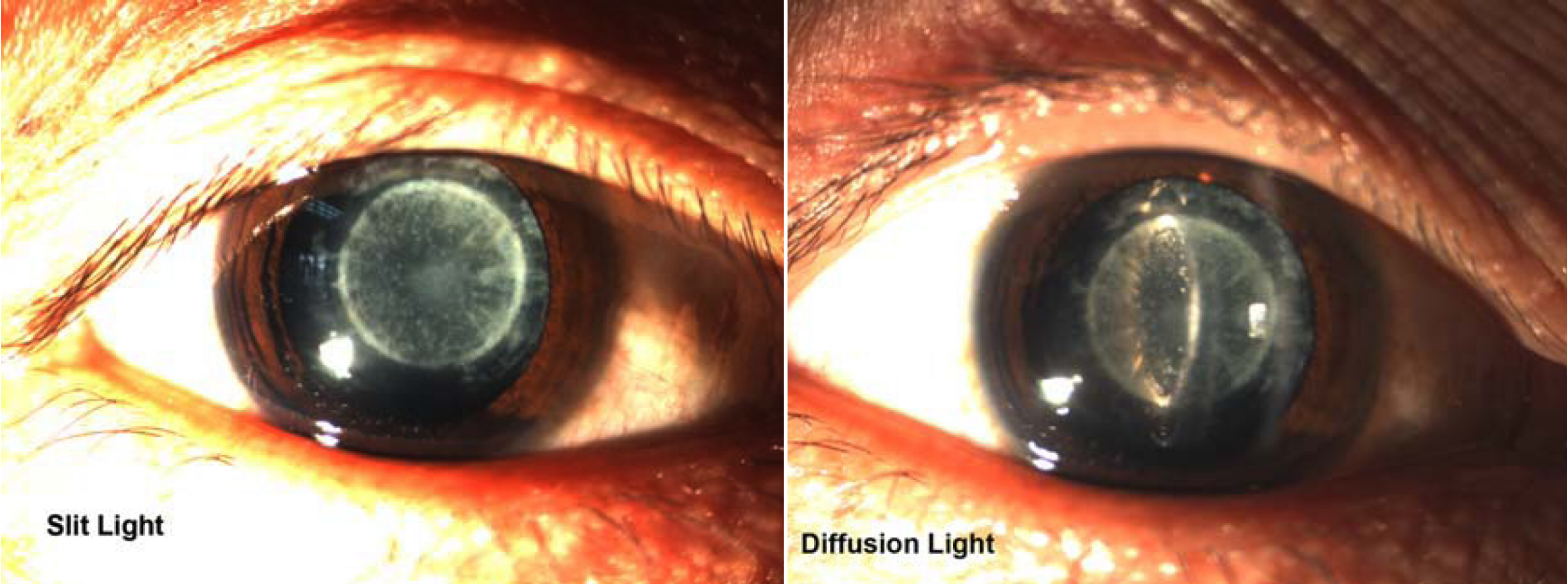
Slit lamp photographs of proband. The photographs of the proband (IV:25) showed that the opacities were lamellar cataracts.

The proband was a six-year-old female (IV:25) who had bilateral cataracts since birth. Her visual acuity was 0.1 in the right eye and 0.08 in the left eye. Binocular vision correction does not improve. Intraocular pressure (IOP) was measured to be 15.1 mmHg in the right eye and 16.7 mmHg in the left eye. Other eye examinations included mydriasis later, slit lamp biological microscopy, and performance of the congenital lamellar cataracts (Figure 1).

**Figure 2:**
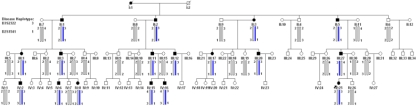
Pedigree and haplotype of cataract family. Four-generation pedigree, with autosomal dominant lamellar cataracts. Haplotyping shows segregation of two microsatellite markers on 3q21-q25. Squares and circles indicate males and females, respectively. Blackened symbols and bars denote affected status.

This four-generation family included 17 affected individuals with congenital lamellar cataracts and 56 unaffected individuals (Figure 2). The diagnosis was confirmed by ophthalmologists. The disease feature of this family is congenital morbidity eyes. There was no history of other ocular or systemic abnormalities in the family.

### Linkage and haplotype analysis

*BFSP2* on chromosome 3 was linked to this family while other candidate genes were excluded by allele sharing and linkage analysis. Significant linkage was found with markers D3S2322 and D3S1541, and the maximum LOD score was 7.22 (at θ=0). Haplotype analysis showed that the responsible locus was localized on chromosome 3q21–25 and flanked by markers D3S2322 and D3S1541 (Figure 2 and Table 2).

**Table 2:** Two-point LOD scores for linkage between cataract locus and markers on chromosome 3.

**Marker**	**LOD scores by recombination fraction (θ)**				
	**0**	**0.1**	**0.2**	**0.3**	**0.4**
D3S2322	7.22	6.08	4.8	3.35	1.72
D3S1541	5.42	4.55	3.58	2.48	1.24

Note: The highest observed LOD score was 7.22 (θ=0) for marker D3S2322.

### Mutation analysis for *BFSP2*

**Figure 3:**
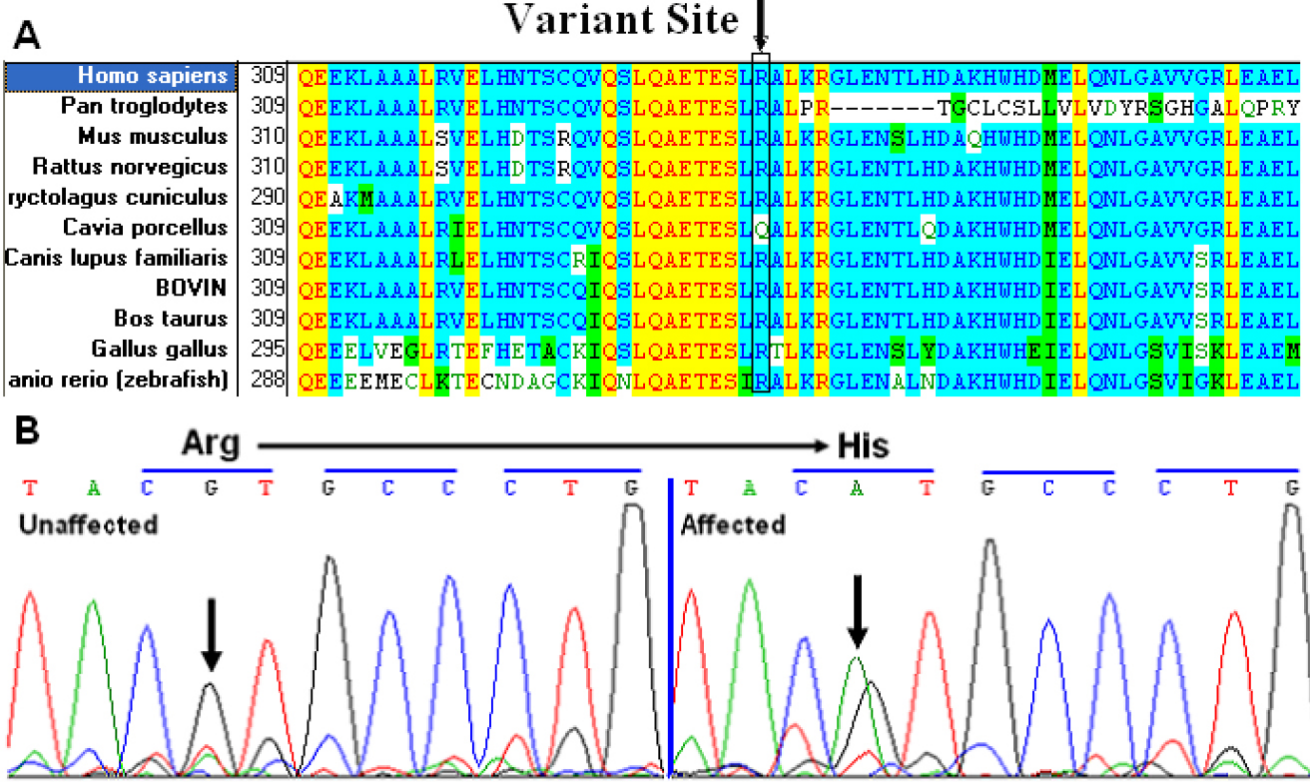
Multiple-sequence alignment and DNA sequence chromatograms of *BFSP2*. **A**: The multiple-sequence alignment of BFSP2 from primates, rodents, cattle, and zebrafish to humans (*Homo sapiens*) is shown. The Arg339 residue is located within a highly conserved region. **B**: DNA sequence chromatograms of the P.R339H mutation in *BFSP2* are shown. The G→A transversion at position 1091 resulted in the P.R339H mutation.

Direct cycle sequencing of the amplified fragments of *BFSP2* in two affected individuals identified a single base alteration, C.G1091A (Figure 3B), in exon 5 of *BFSP2* (GI: 8419), resulting in the substitution of Arg to His at codon 339 (P.R339H). The remainder of the coding sequence showed no other change.

### Multiple-sequence alignment and mutation analysis

BFSP2 family protein sequences were obtained from NCBI and UCSC websites and multiple-sequence alignments of BFSP2 family proteins from various species were obtained (Figure 3A) using the Vector NTI software. We found that codon 339, where the mutation (P.R339H) occurred, was located in a highly conserved region of the protein.

### Denaturing high performance liquid chromatography

**Figure 4:**
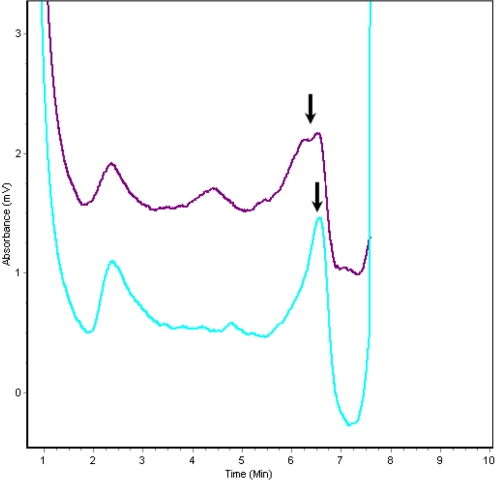
Denaturing high-performance liquid chromatography results of wild type and mutated *BFSP2*. DHPLC results show a variant trace for *BFSP2* compared to the wild type (WT) trace. The profile in purple is the mutant protein. The profile in blue is the wild type protein.

DHPLC analysis confirmed this mutation (Figure 4), which cosegregated with all affected individuals in the family. Further, this mutation was not observed in any of the unaffected family members or 100 normal controls.

## Discussion

We identified a new mutation, P.R339H, in *BFSP2* in a four-generation Chinese family with autosomal dominant congenital lamellar cataracts. The disease gene was linked to 3q21–25 with a maximum LOD score of 7.22 and spanning *BFSP2*. Mutation analysis of the candidate gene detected a new mutation, P.R339H, in *BFSP2* that cosegregated with the disease phenotype in all affected individuals but was not present in any of the unaffected family members or in 100 normal control subjects. The result of multiple-sequence alignments showed that Arg339 was a highly conserved residue.

The lens sutures are specific regions where lens fiber cells from opposite directions merge through complex overlapping and interdigitation of the tips of their membranes [24]. The beaded filament structural protein encoded by *BFSP2* is a highly divergent member of the intermediate filament family and a major component of beaded filaments, which are abundant in lens fiber cells, the only cells in which they are known to be expressed. These cytoskeletal structures consist of a 7-9 nm backbone filament with 12-15 nm globular protein particles spaced along it [16]. During lens development, the anterior ends of the lens fiber cells progressively insinuate between the lens epithelium and the embryonic nucleus whereas the posterior ends progressively insinuate between the posterior lens capsule and embryonic nucleus. Elongation continues until the fiber cell ends from opposing directions meet to form the lens sutures [25].

Through direct cycle sequencing, we identified a G→A transversion in exon 5 of *BFSP2*, which was present only in affected members of the family. This transversion, C.G1091A, was predicted to cause a conservative substitution of Arg to His at codon 339 (p.R339H). This novel mutation in exon 5 of *BFSP2* appears to result in congenital lamellar cataracts.

**Figure 5:**
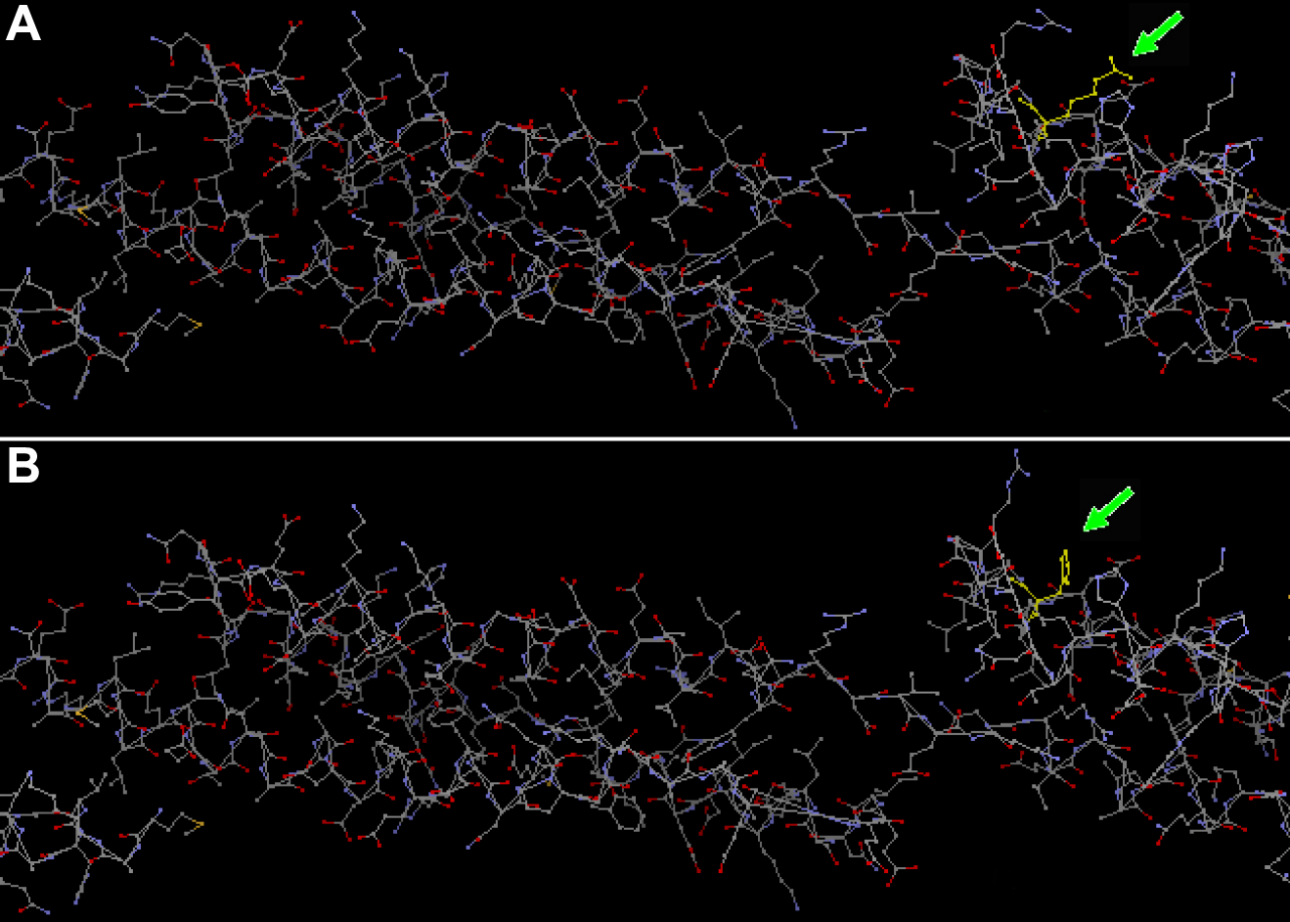
Comparison of wild type and mutant BFSP2 3D structures. **A**: Wild type BFSP2 3D structure using Phyre software is shown, and Arg339 is indicated in yellow and a green arrow. **B**: Mutant BFSP2 3D structure is shown also using Phyre software, and His339 is indicated in yellow and a green arrow. The 3D structure was not overly changed due to the mutation.

**Figure 6:**
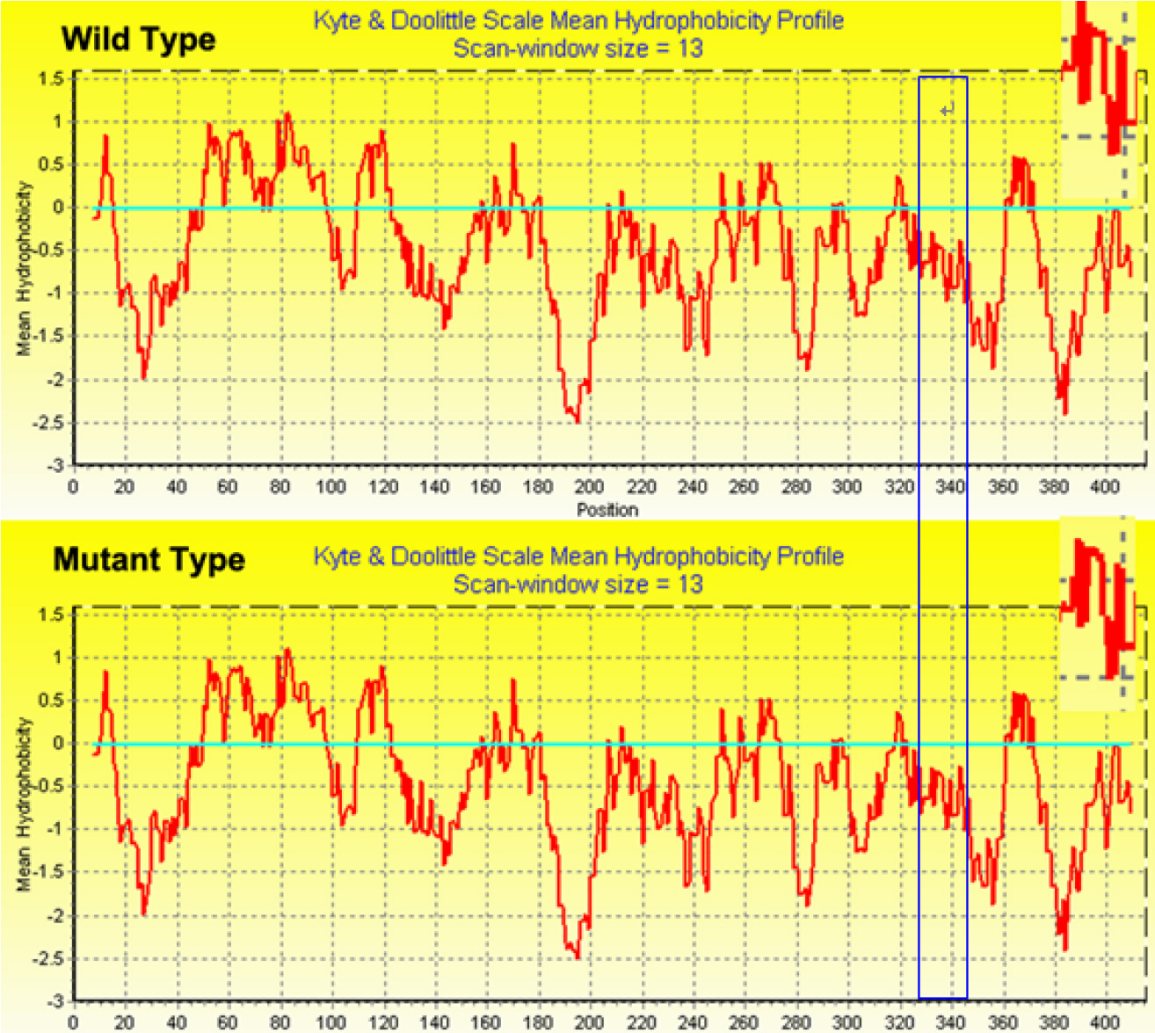
Comparison of hydrophobicity between wild type and mutant BFSP2. BioEdit software predicted the effect of the substitution on BFSP2 protein hydrophobicity. Hydrophobicity of the mutant protein increased between 330 amino acids and 340 amino acids.

**Figure 7:**
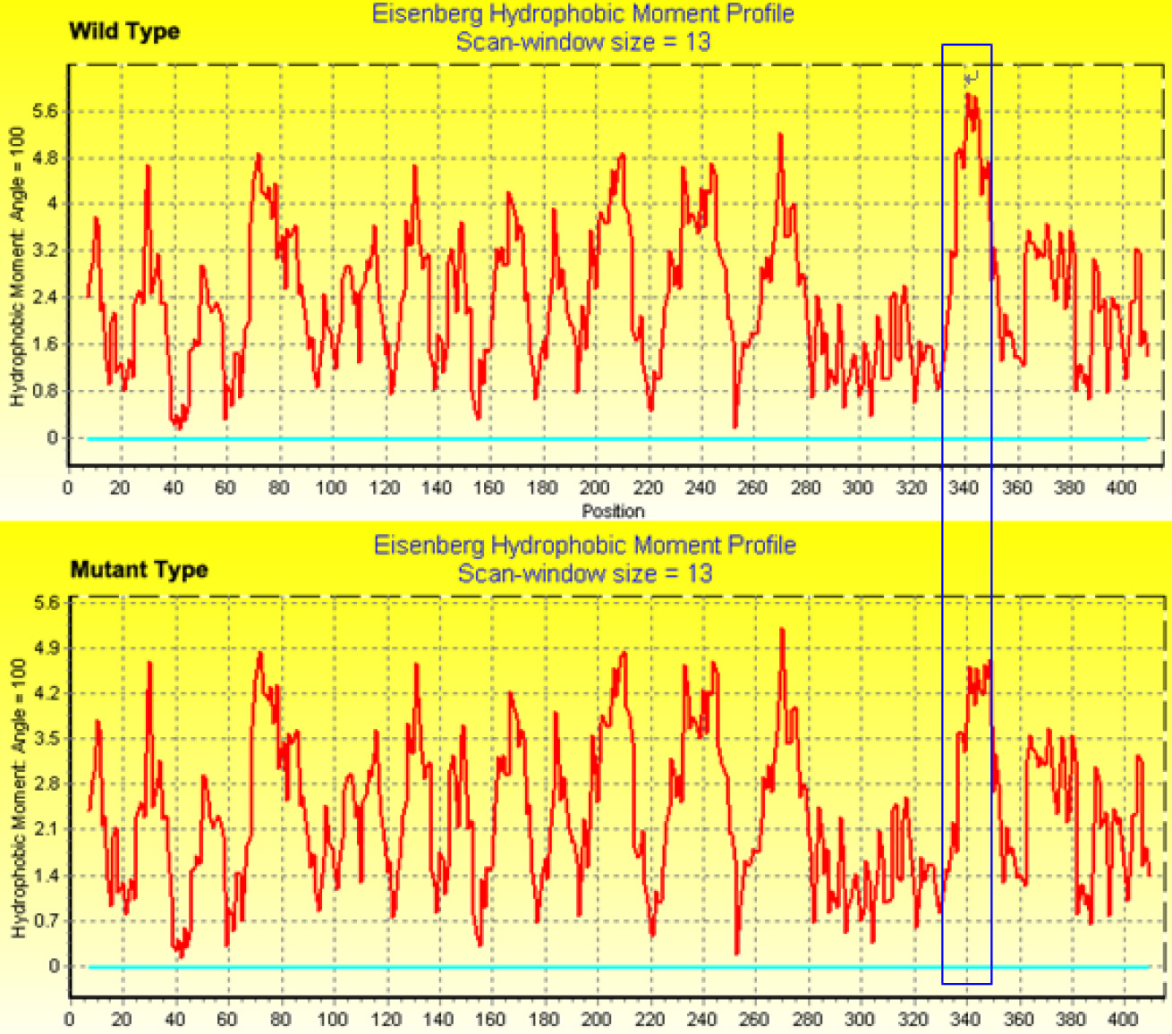
Comparison of hydrophobic moment between wild type and mutant BFSP2. BioEdit software predicted the effect of the substitution on the BFSP2 protein hydrophobic moment. The hydrophobic moment of the mutant protein decreased between 330 amino acids and 350 amino acids.

From the NCBI database, we know Arg339 is in the active region of the BFSP2 protein. The online Phyre software (version 0.2) was used to compare the three dimensional (3D) structures of the wild type (Figure 5A) with mutant proteins (Figure 5B), and the 3D structure was little changed. BioEdit software predicted that the substitution in the BFSP2 protein would alter the hydrophobicity between amino acids 330 and 340 (Figure 6) and the hydrophobic moment between amino acids 330 and 350 (Figure 7).

**Figure 8:**
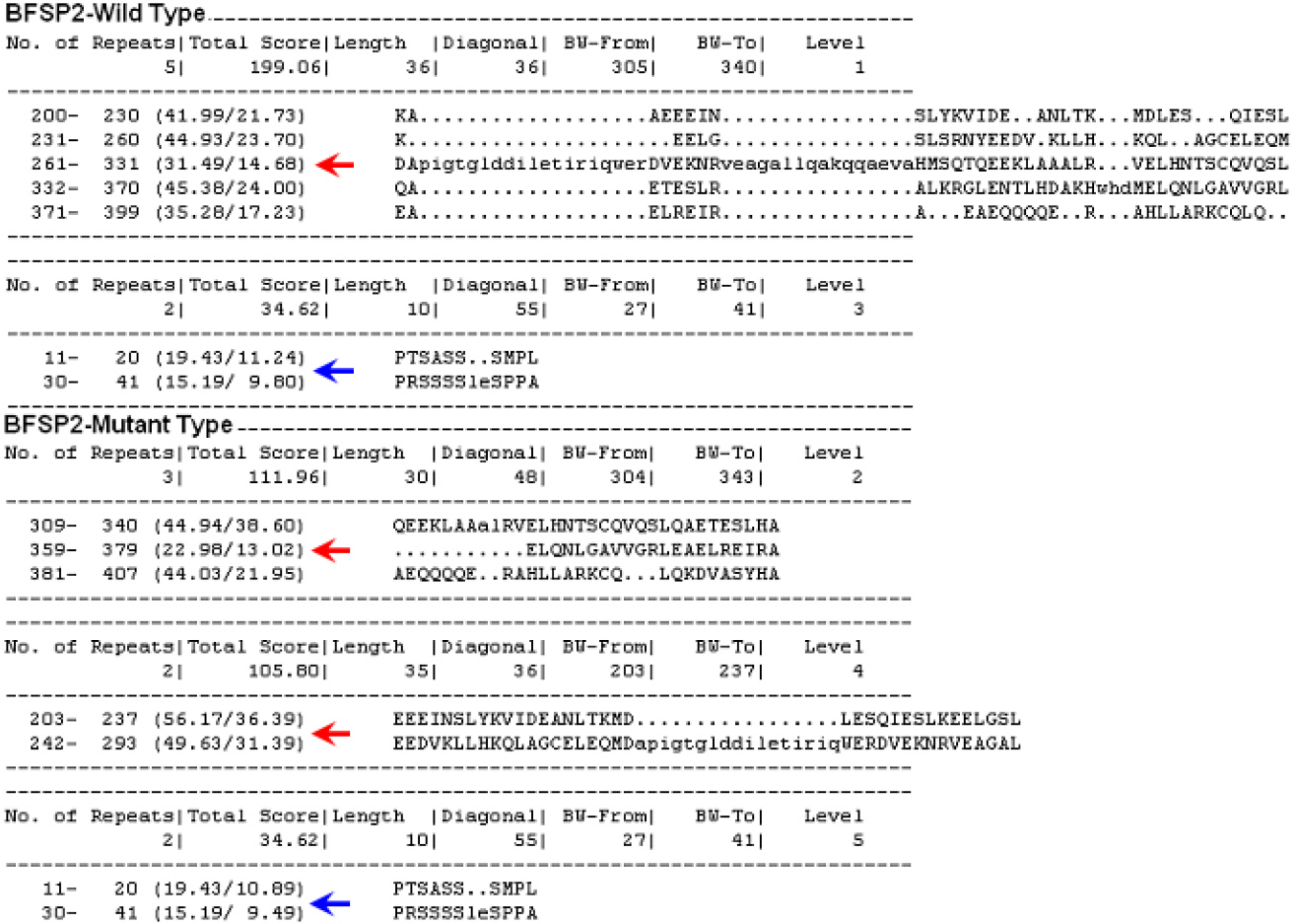
The predicted effect of substitution in BFSP2. The online Radar software was used to predict the effect of the substation in BFSP2. The substitution would have in the wild-type protein with an increase from two to three repeats, the part marked by red arrow turned into two parts.

Furthermore, we used Radar to predict the effect that the substitution would have in the wild type protein with an increase of two to three repeats (Figure 8). Many large proteins have evolved by internal duplication, and many internal sequence repeats correspond to functional and structural units.

The mechanism of how a mutation in *BFSP2* can give rise to cataracts is unknown. BFSP2 shows most of the secondary structural features that are conserved among the intermediate filament (IF) family of proteins [26]. Some reports indicate that BFSP2, filensin, and α-crystallins have been shown to immunolocalize to unique cytoskeletal structures within the lens fiber cells known as beaded filaments. Beaded filaments are considered to be important in facilitating the chaperone activity of α-crystallin assemblies [6,27]. Some reports show that BFSP2 can regulate beaded filament protein stoichiometry [28].

The alteration had little effect on the backbone or 3D structure of the protein. Hydrophobicity of the mutant protein increased while the hydrophobic moment decreased. The predicted new characteristics of the mutant protein, which include altered interactions with other proteins, altered regulation activities of BFSP2, and altered α-crystallin assemblies, may be the cause of the disease.
